# Magnetic capture device for large volume sample analysis

**DOI:** 10.1371/journal.pone.0297806

**Published:** 2024-02-09

**Authors:** Cheryl M. Armstrong, Joseph A. Capobianco, Joe Lee

**Affiliations:** United States Department of Agriculture, Agriculture Research Service, Eastern Regional Research Center, Wyndmoor, Pennsylvania, United States of America; Nelson Mandela African Institute of Science and Technology, UNITED REPUBLIC OF TANZANIA

## Abstract

Immunomagnetic separation (IMS) techniques employing superparamagnetic particles can successfully isolate various components from mixtures. However, their utility can be limited for large-volume samples, viscous samples, or those containing a high density of particulate matter because of the need to generate high field gradients for particle recovery. Therefore, a new class of immunomagnetic particles was devised utilizing a single, macroscopic Pyrex spinbar conjugated with biorecognition elements to address these limitations. Advantages include an inherent capacity for effective mixing, an almost instantaneous recovery of the spinbar that can be performed without expensive equipment and with no loss of magnetic particles during processing, and reduced transfer of sample matrix. As a result, spinbars can provide an effective means for IMS with large-volume assays composed of complex matrices.

## Introduction

Transforming samples from their native form to one that is suitable for analysis is key to the success of many diagnostic assays. Techniques applied for sample transformation can range from chemical modifications (sample preparation) to physical modifications (sample pretreatments) of the sample. Reviews covering many of the different methods currently available can be found the scientific literature [1–3]. One common technique used in conjunction with mainstream diagnostic assays is known as immunomagnetic separation (IMS).

IMS employing the use of superparamagnetic particles (SPPs) for the isolation/purification of specific targets from heterogeneous mixtures has become standard practice in the field of molecular biology. Multiple methods have been developed for preparing the small magnetic microspheres commonly used for IMS. The chemical synthetic method developed by Ugelstad in 1970s remains the most successful route and forms the basis of the commercialized SPPs [4]. Monosized particles that have surfaces amendable to conjugation with target-specific biorecognition elements while also displaying resistance to swelling and overall degradation are highly desirable for this purpose because they balance the need for both function and storage [5, 6]. Additionally, the lack of remanence in SPPs ensures their magnetic properties are only apparent when an external magnetic field is applied [7]. Together, these features allow the SPPs to be homogeneously dispersed throughout the sample suspension in the absence of a magnetic field and increases their likelihood of contacting/capturing targets. After incubation with a sample, recovery of the SPPs can be facilitated with a magnet. This not only aids with washing but can minimize elution volumes as well.

Multiple factors affect the ability of these particles to capture their targets including particle construction, size, and sample composition. Current commercialized SPPs are small, ranging from micrometers to nanometers in size. Some studies have suggested that smaller particles improve performance by both increasing the number of particles that can bind to a given target and by providing an increased surface area upon which binding can occur [8, 9]. However, these studies were performed with the capture being conducted in small sample volumes (<1 mL) and therefore may not be representative of the outcomes in large sample volumes. With large volume samples, mass transport [10], collision probability [11], and magnetic recovery [6] may be the main factors influencing recovery. Comparison studies conducted with beads of various sizes and densities concluded that larger/ heavier beads captured bacteria more effectively than smaller/ lighter beads in larger volumes [12–14]. This was because bead sedimentation directly correlated with sample volume traversed, which resulted in increased interactions amongst beads and bacterial cells [12–14]. Magnetic recovery performance may also be enhanced with larger particles. However, size and density alterations can contradict the ability of SPPs to remain homogenously distributed; a property that effects bead-target interactions.

The interplay of these factors along with others, such as time and cost, must be assessed to determine if a functional assay can be developed. For example, interaction rates between SPPs and targets decrease as sample volumes increase, when all variable aside from sample volume are kept constant. Because of this, additional particles are required as volumes increase if an equivalent number of targets are to be captured in an equivalent time. If particle number is not increased, then longer assay times are needed, which may ultimately lead to assay times or particle costs becoming prohibitive. Moreover, sample composition must also be considered during assay development since samples that are highly viscous or those that contain an abundance of heterogeneous particulate matter can hinder dispersion and/or recovery of SPPs [6, 15, 16]. Batch-based adsorption has been adopted in many industrial applications involving bio-purification via magnetic separation because it allows for large volumes of more complex matrices to be assayed [17, 18]. Although effective, complicated and sometimes expensive processing equipment must be employed to recapture the microscopic-sized magnetic particles currently used in these industrial applications. This can be seen with the high gradient magnetic separator-based devices utilized for high-gradient magnetic fishing [19]. Biofouling of such equipment may present additional obstacles when dealing with certain sample types.

Here we report on a novel method to help overcome many of the limitations associated with IMS techniques and challenge the notion that microscopic magnetic particles are necessary for efficient IMS. This simple method uses a single 12.7 millimeter-long Pyrex Spinbar coated with biorecognition elements and a low-tech magnetic stir plate for effective IMS with large-volume samples composed of complex matrices.

## Materials and methods

### Bacterial strains and plasmid construction

The pET-6XHis/TEV/EGFP, a plasmid encoding a 6X His tag followed by a TEV protease site and the enhanced green fluorescent protein (EGFP) gene, was purchased from Vector Builder (Chicago, IL). The plasmid was transformed into *Escherichia coli* BL21(DE3) (Invitrogen, Waltham, MA) for the inducible expression of EGFP. *E*. *coli* O157:H7-PC [20] was also utilized for cell capture studies since the anti-*E*. *coli* antibodies used in this study have been shown to be effective for its capture [21]. Luria-Bertani medium (LB) (BD Difco), LB containing 50 μg/mL ampicillin sodium salt (Fisher Scientific, Fair Lawn, NJ), or LB containing 100 μg/mL of spectinomycin dihydrochloride pentahydrate (GoldBio.com, St. Louis, MO) was used for the growth and maintenance of the *E*. *coli* strains within this study. Overnight cultures of *E*. *coli* were grown at 37°C with shaking at 180 rpm for ~17 hr.

### Surface functionalization of the Pyrex spinbars

Pyrex^®^ Spinbars (Bel-Art Products, Wayne, NJ) were chosen for this application because of the well-established protocols for the functionalization/conjugation of glass [22–26], allowing the deposition of antibodies onto the spinbar. For these assays, either affinity purified BacTrace ^®^ goat anti-E. coli O157:H7 antibodies (Seracare, Milford, MA) or anti-His Tag (HIS.H8) monoclonal antibodies (Epitope Biotech Inc., Vancouver, British Columbia) were deposited onto 12.7 X 9.5 mm Pyrex^®^ Spinbars following the surface treatment described here. Initially, spinbars were treated with piranha solution to remove any organic matter and hydroxylate the surface. The piranha solution was prepared by mixing a 3:1 volume ratio of reagent grade 96% sulfuric acid (Sigma Aldrich, St. Louis, MO) and 30% hydrogen peroxide (Sigma Aldrich). The surfaces were then thoroughly rinsed in water, anhydrous alcohol, and air dried under ambient conditions.

3-Mercaptopropyltriethoxysilane (MPTS) was deposited onto the spinbars using methods previously described [27]. Briefly, the glass surfaces were submerged in a 1% MPTS solution in 95% ethanol titrated to a pH of 4.5 using acetic acid. After an overnight incubation, surfaces were sequentially rinsed twice with 95% ethanol titrated to a pH of 4.5 using acetic acid, Nanopure water, and then cured for 3 days at room temperature.

Antibodies were immobilized to the thiol group on the glass surface using a heterobifunctional crosslinker sulfo-(succinimidyl 4-[N-maleimidomethyl]cyclohexane-1- carboxylate) (Sulfo-SMCC) according to the manufacturer’s guidelines. Briefly, antibodies were diluted to a concentration of 1x10^-6^ g/mL. Sulfa-SMCC is then reacted with antibodies with a 20-molar fold excess and allowed to react for 30 min at room temperature. After 30 min, unreacted SMCC was removed using a 7000 MW Zebra Spin column (Thermo Fisher Scientific, Rockford, IL). The Sulfo-SMCC activated antibodies are applied to the MPS-coated spinbars in a manner that ensures 30X the number of antibodies required to densely pack on the surface (~5x10^13^ antibodies/spinbar). The spinbars are allowed to incubate for at least 1 hr at room temperature. Prior to use, the spinbars were rinsed with phosphate buffered saline (PBS) containing 0.05% Tween-20 (140 mM NaCl, 3 mM KCl, 0.05% TWEEN 20 Detergent, 10 mM phosphate buffer, pH 7.4) (Sigma Aldrich).

### Verification of antibody immobilization

Pyrex spinbars that had undergone the surface functionalization process with 6X His mAB/HRP conjugate (TaKaRa Cat # 631210 Lot # 1604468A) were used to verify the coupling of the antibodies to the surface of the spinbar. For this, after functionalization, spinbars were washed individually in 5 mL PBS in 15 mL conical tubes and stored at 4°C till use. A 3,3′,5,5′-tetramethylbenzidine (TMB) /H_2_O_2_ solution was prepared using a 0.3 mM TMB solution that was derived from a stock containing 6 mg of TMB mixed with 4 mL acetonitrile and subsequently diluted in a 0.20% sodium acetate buffer containing 15 mL of acetonitrile (pH 4.8) as previously described [28]. The day of the experiment, 64 μL of 3% hydrogen peroxide was added to 10 mL of the prepared TMB solution to produce the TMB/ H_2_O_2_ solution. For the verification assay, each PBS-washed spinbar was transferred into a sterile NEST Scientific 3.5 mL polypropylene, 48-deep well, U-bottom, round well plate (Stellar Scientific, Baltimore, MD). Spinbars were covered with 500 μL of the TMB/ H_2_O_2_ solution, gently agitated via softly swirling the plate, and then incubated statically at room temperature in the dark for 20 min. The enzymatic assay was then stopped using 500 μL of 1 M sulfuric acid. Spinbars that did not have antibodies immobilized upon their surface were subjected to identical conditions for comparison purposes. Positive controls consisted of 0.3 μL of the 6X His mAB/HRP diluted to 1x10^-6^ g/mL, whereas negative controls were TMB/ H_2_O_2_ solution alone. Results of the oxidative reaction were measured via absorbance at 450 nm on a Tecan Safire2 plate reader (Tecan Group Ltd.; Männedorf, Switzerland).

The ability of antibodies conjugated to the surface of the spinbar to retain their functional activity was verified via confocal microscopy. Microscopic images were obtained by first placing spinbars conjugated with anti-His Tag (HIS.H8) monoclonal antibodies in a solution containing purified green fluorescent protein (GFP) that had been modified to include a 6X HIS tag (Abcam, Waltham, MA). Mixing was performed at 350 rpms for 10 min on a Digital Magnetic Hotplate Stirrer Pro (VWR International, Radnor, PA). Excess GFP was removed from the spinbar’s surface by placing the spinbar in 30 mL of a 20 mM Tris-HCl; pH 7.5 wash solution and agitated for 2 min. Samples were viewed on a Leica DMI4000B confocal microscope with a 40X objective (Leica Microsystems, Mannheim, Germany). Images were captured using Leica LAS-X software. Excitation and emission wavelengths were 488 nm and 509 nm, respectively. As a control, spinbars not exposed to GFP were also imaged.

### Surface functionalization of SPPs

Tosylactivated Dynabeads M-280 were conjugated with the same antibodies as the spinbars using the manufacturer’s suggested protocol. Briefly, 10 mg of beads was pelleted, decanted, and washed twice in 0.1 M sodium phosphate buffer. The beads were then conjugated using 0.2 mg of antibody in 250 μL of 1.2 M ammonium sulphate buffer. The suspension was vortexed and incubated at 37°C on a rotatory mixer (Dynal Biotech, Lake Success, NY) set to 25 for 18 hrs. The solution was decanted and replaced with 1 mL of 0.5%w/v BSA in PBS. The suspension was vortexed and incubated at 37°C on a Dynal Biotech rotatory mixer set to 25 for 2 hrs. Finally, the solution was decanted and the pellet was dispersed in 500 mL of 0.1% BSA in PBS to ensure a bead concentration of 20 mg/mL.

Dynabeads were assumed to have a diameter of 2.8 μm [29] and a density of 1.8 g/cc [30, 31]. These assumptions were used to estimate that 2.12x10^7^ Dynabeads had an equivalent surface area to the 12.7 X 9.5 mm spinbar. The weight/volume concentration of the Dynabeads was used to calculate the volume of beads needed for each assay.

### Capture of enhanced green fluorescent protein

Expression of EGFP was achieved by inoculating 500 mL of sterile LB medium containing 50 μg/mL of ampicillin with 5 mL of an overnight culture of *E*. *coli* BL21(DE3) + pET-6XHis/TEV/eGFP. Cells were grown to midlog phase (OD_600_~0.6) at 30°C with shaking at 160 rpm and EGFP expression was induced through the addition of 0.5 mL of 1M isopropyl β-d-1-thiogalactopyranoside (IPTG) (Fisher Scientific). After an additional ~1.75 hr of growth post induction, ~250 mL aliquots of cells were pelleted by centrifugation at 5000 X g for 10 min at 4°C. Pellet weights were noted and cells were frozen at -80°C for at least 30 min. To ensure efficient lysis and eliminate nucleic acids that could interfere with protein binding, cell pellets were thawed at roomed temperature and subsequently resuspended in Complete Bacterial Protein Extraction Reagent (B-PER) (Thermo Scientific, Rockford, IL) containing Pierce Complete, mini, EDTA-free Protease inhibitor Cocktail (Thermo Scientific). B-PER was added at a rate of 4 mL per gram of pelleted cells and protease inhibitors at a rate of 1 tablet per 10 mL of B-PER as per the manufacturers’ instructions. An identical procedure was followed using *E*. *coli* BL21(DE3) for use as a control except ampicillin was not added to the LB.

Cell lysates (4–6 mL) were placed into 1 L glass media containers. The volume of the solution was brought up to 500 mL using 20 mM Tris-HCl; pH 7.5. Spinbars or SPPs coated with anti-8X His antibodies were warmed to room temperature, added to the appropriate cell lysate with the media containers being subsequently capped, and mixing occurred either at 350 rpms for 10 min on a Digital Magnetic Hotplate Stirrer Pro (spinbars) or by shaking the container at room temp for 10 min at 120 rpm (SPPs). To capture the SPPs for washing purposes, magnets were taped to the outside of the media container and the containers were laid down so that the magnets were positioned at the bottom of the media container. The media bottles then remained in this static position for 30 min to ensure maximum capture of the SPPs. After 30 min, the media bottles were gently rotated so that the magnetic strip was now positioned along the top of the container and the cell lysate was carefully dumped from the container. To remove any loosely bound material, SPPs were washed in 30 mL of 20 mM Tris-HCl; pH 7.5 using gentle circular agitation with the magnets firmly affixed to the media bottles. Media bottles were allowed to stand for 5 min without agitation to ensure capture of the SPPs and the wash solution was then dumped from the media bottle. Magnets were removed from the media bottles and 15 mL of 20 mM Tris-HCl; pH 7.5 was added to resuspend the SPPs. Bottles were swirled to help release the beads with all the solution subsequently passing through a MACS Large Cell Separation Column (Miltenyi Biotec, Germany) placed against a magnet using gravity flow to maximize SPP recovery. Upon removal of the MACS column from the magnet, plunging of the column was performed with 350 μL of 1X Tobacco Etch Virus (TEV) protease buffer (50 mM Tris, 0.5 mM EDTA, 1 mM DTT, pH 8.0).

To wash the spinbars, magnets were used to capture the spinbar against the lid of the media bottles to allow for transfer of the spinbars to a clean 50 mL conical tube containing 30 mL of 20 mM Tris-HCl; pH 7.5 wash solution. Spinbars were agitated for 2 min in the wash solution. They were subsequently transferred to a sterile NEST Scientific 3.5 mL polypropylene plate where they were covered with 350 μL of 1X TEV protease buffer. It is worth noting that spinbars were not touched throughout the duration of the experiment. All transfers were made by placing a magnet on the outside of a container’s lid, exploiting the magnetic forces for movements of the spinbars.

### Cleavage of EGFP from spinbar/ SPPs

After washing, EGFP was cleaved from the spinbars/ SPPs though the TEV protease site located between the His tag and EGFP. Spinbars/SPPs placed in the 350 uL of 1X TEV buffer had 7 uL of rvTEV Protease (R&D Systems, Minneapolis, MN) added to each well and the plate was incubated statically at 30°C for 1 hr. 100 uL of the reaction volume was then added to a clean Corning Costar Assay plate, 96-well non-treated black with clear bottom (Sigma Aldrich). Fluorescence was measured from the bottom on a Tecan Safire 2 at 483 nm excitation/ 525 nm emission with the gain setpoint at 51.

### Capture and detection of *E*. *coli* O157:H7

*E*. *coli* O157:H7-PC was grown overnight at 37°C with agitation in LB broth containing 100 μg/mL of spectinomycin. The next day a 1:1,000 and a 1:10,000 dilution of the overnight was made in 500 mL modified Tryptic Soy Broth (mTSB) (Sigma Aldrich) in glass 1 L media bottles. A 350 μL aliquot was removed from each diluted culture for use as a positive control for the PCR analysis and two aliquots (1 μL or 10 μL) were plated onto LB plates containing 100 μg/mL of spectinomycin in order to quantify the number of live cells within each diluted culture. It is worth noting that because of known inhibitors present in ground beef (such as hemoglobin, glycogen and fats [32–34]) and the fact that ground beef is not sterile (therefore it contains microflora that can interfere with cell counts), both the positive control utilized for the PCR and that plated to obtain initial inoculum levels consisted of cells diluted in mTSB prior to the addition of the ground beef.

To accommodate the limited size of the stomacher bags utilized, 166.67 grams of 80% lean ground beef (80/20) that was purchased from a local grocery store was initially added to 150 mL of mTSB inoculated culture in a 177 mm x 305 mm stomacher bag (Fisher). Samples were mixed using the Stomacher 400 Circulator (Seward Labs, England) for 2 min at medium speed. Eluate from the filter side of the stomacher bag was then added to the remainder of the inoculated mTSB culture and swirled to mix. The process was repeated for the no cells control except the mTSB utilized was not inoculated. Pyrex spinbars containing anti-*E*. *coli* antibodies were placed into the 1 L media bottles containing the inoculated ground beef and spun at 350 rpm for 10 min on a Digital Magnetic Hotplate Stirrer Pro (VWR International) while the M-280 Tosylactivated Dynabeads containing anti-*E*. *coli* antibodies were shaken at room temp for 10 min at 120 rpm. Antibody-free spinbars and Dynabeads that had been coated in bovine serum albumin (Sigma-Aldrich, St. Louis MO) were used as additional controls. Washes of the spinbars/SPPs were performed as described above using 30 mL of 1X phosphate buffered saline (pH 7.4) (Boston BioProducts, Ashland, MA) with movement of the spinbars being manipulated via additional magnets.

### Quantification of captured *E*. *coli* via quantitative real-time polymerase chain reaction (qPCR)

Spinbars were transferred from the wash solution to a sterile NEST Scientific 3.5 mL polypropylene plate where they were covered with 350 μL of nuclease-free water. SPPs were eluted into the same plate from the MACS columns using 350 μL of nuclease-free water. Because a cleavable linkage was not present on the antibodies to release the cells from the SPPs or the spinbars, captured cells were lysed and the resulting supernatant was used for qPCR. Lysis of captured cells was performed by boiling the spinbars/SPPs at 95°C for 10 min and then immediately chilling the samples on ice. Lysates were transferred to sterile microfuge tubes and cell debris was subsequently removed via centrifugation at 10,000 X g for 5 min at 4°C. Supernatants were placed into clean microfuge tubes and was utilized for qPCR using the previously described STEC-Shuffle primer/probe set [20]. (This primer/probe combination amplifies a genomic marker placed into the *E*. *coli* O157:H7-PC strain that was utilized in these experiments and allows it to be differentiated from other *E*. *coli* strains that may be present naturally in the ground beef matrix utilized.) For qPCR, 8 μL of the supernatant was placed into a MicroAmp Fast Reaction tube (Applied Biosystems) along with 10 μL of Dynamo Flash master mix (Thermo Fisher), 0.5 μL of STEC-Shuffle-F (20 μM), 0.5 μL of STEC-Shuffle-R (20 μM), 0.25 μL of STEC-Shuffle-P (20 μM), 0.4 μL of 50X ROX and 0.35 μL of nuclease-free water. Conditions utilized for qPCR were identical to those reported by Paoli, *et*. al [20]. Ct values were reported using the StepOnePlus Real-time PCR system and software v 2.3 (Thermo Fisher).

### Statistical analysis

**Fig 1:**
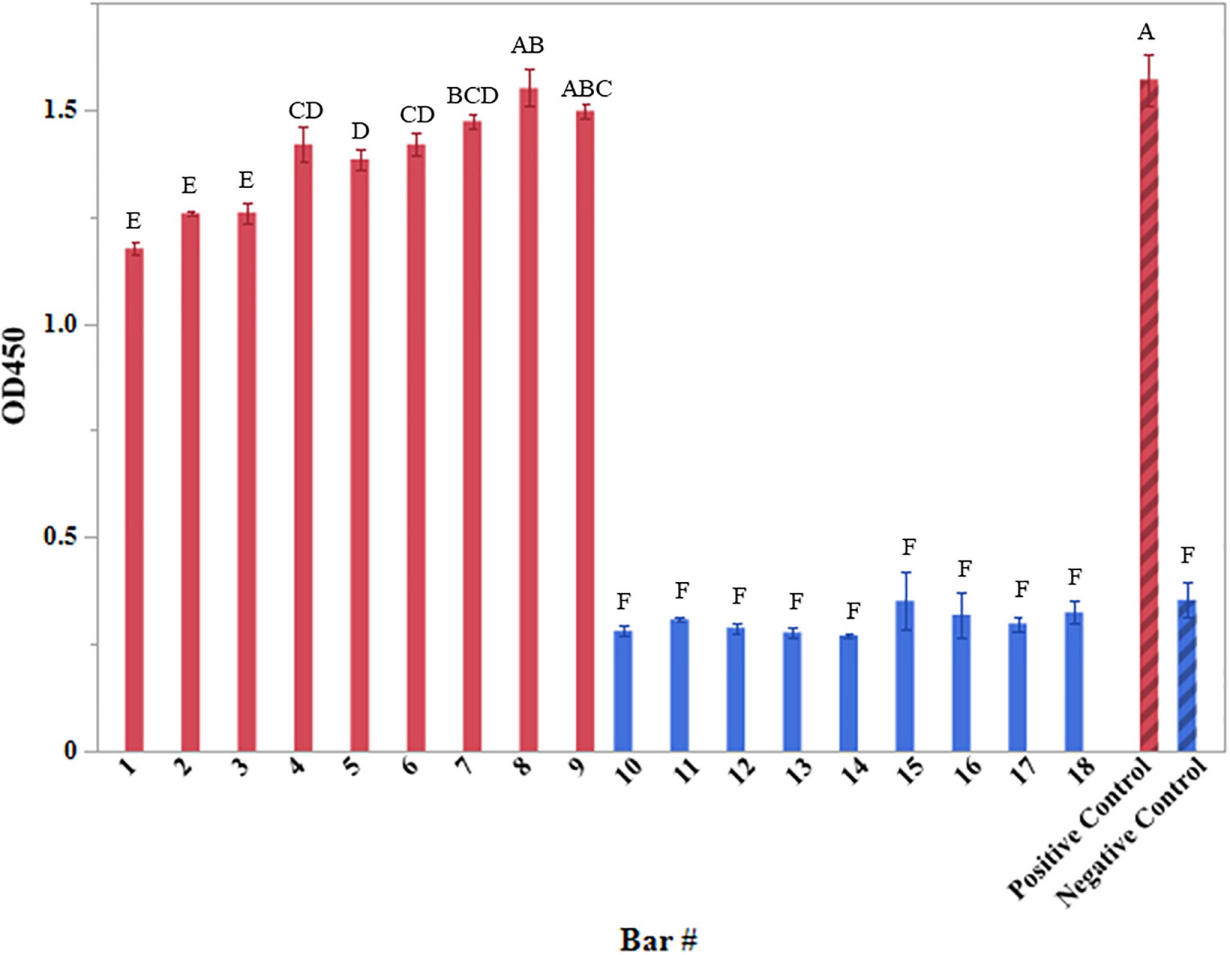
Immobilization of antibodies on spinbars. Pyrex-coated spinbars both with anti-His antibodies conjugated to HRP (red) and without antibodies (blue) were tested for the presence of HRP via an enzymatic assay that results in the oxidation of TMB and production of a chromogenic product. The chromogenic product was measured via spectrophotometry at 450 nm. The positive control (striped, red bar) contained purified HRP-conjugated antibodies and the negative control (striped, blue bar) contained the TMB/H_2_O_2_ alone, with neither control containing a spinbar. Averages from 3 readings are shown with error bars representing the standard deviation of the mean. Groups containing different letter designations were deemed significantly different (p≤ 0.05).

**Fig 2:**
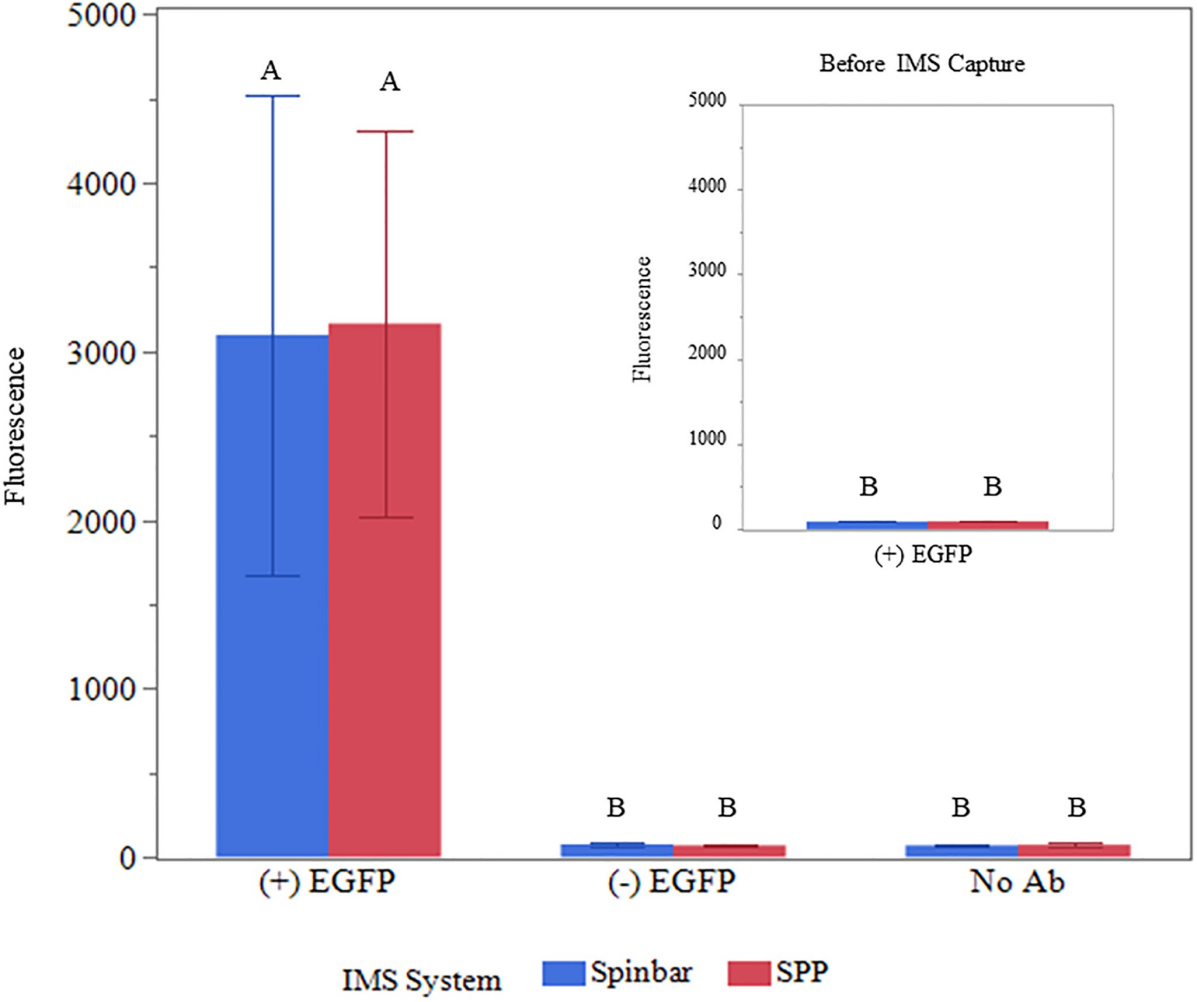
IMS using Pyrex spinbars and SPPs to capture protein. Assessment of the capture of His-tagged EGFP using spinbars (blue bars) or SPPs (red bars) coated with antibodies specific for His in 0.5 L cell lysate solutions. Fluorescence measurements for spinbars and SPPs coated with 8X His antibodies were obtained from bacterial cultures that expressed EGFP (+ EGFP) and those that did not (- EGFP). Non-conjugated spinbars and SPPs (no Ab) were also assessed for capture in the presence of an EGFP expressing bacterial culture as a control. Inset displays the background fluorescence of EGFP expressing bacterial culture solutions. Averages from 3 independent trials are shown with error bars representing the standard deviation of the mean. Groups containing different letter designations were deemed significantly different (p≤ 0.05).

**Fig 3:**
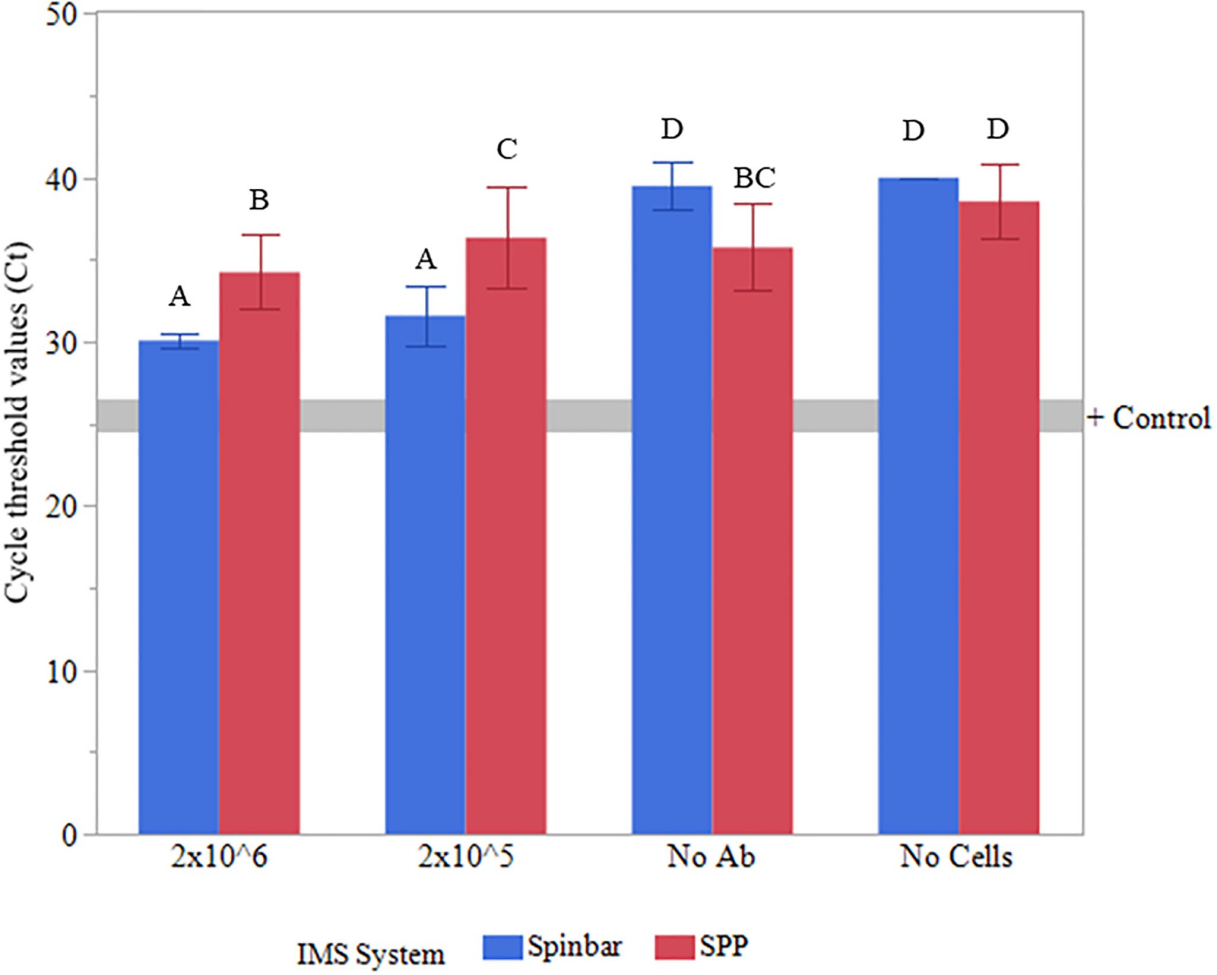
Cell capture via IMS using Pyrex spinbars and SPPs. Assessment of *E*. *coli* cells captured by the anti-*E*. *coli* conjugated spinbars (blue bars) or anti-*E*. *coli* conjugated SPPs (red bars) from a 0.5 L ground beef slurry using qPCR. Non-inoculated ground beef samples (no cells), two inoculation levels of *E*. *coli* O157:H7 (10^5 and 10^6 cells), and spinbars/SPPs without antibodies (no Ab) in the presence of 10^6 *E*. *coli* O157:H7 cells were compared. Averages from 3 independent trials are shown with error bars representing the standard deviation of the mean. The grey line represents the Ct value obtained from *E*. *coli* without ground beef present for use as a positive control. Groups containing different letter designations were deemed significantly different (p≤ 0.05).

All quantitative data was analyzed and graphically presented using JMP software version 16.2.0. Three independent replicates, with each replicate consisting of 3 spinbars, whose chromogenic product was measured in triplicate, were performed for Fig 1. The average of 3 independent trials are also shown in Figs 2 and 3 with error bars representing the standard deviation of the mean in Figs 1–3. Results from Tukey’s honestly significant difference (HSD) test are also shown with groups containing different letter designation determined to be significantly different (p≤ 0.05).

## Results

To verify that antibodies could be immobilized on the surface of the Pyrex-coated spinbars, a validation experiment was performed using anti-HIS monoclonal antibodies conjugated with horse radish peroxidase (HRP) (Fig 1). Spinbars that had undergone the surface functionalization process to allow the immobilization of the HRP-conjugated monoclonal antibodies were compared to those that did not undergo the process. The assessment assayed for the presence of HRP via an enzymatic reaction that resulted in the oxidation of TMB to produce a chromogenic product. On average, spinbars that underwent the surface functionalization process exhibited absorbance readings that were ≥4.6-fold greater than those that did not. This confirms the presence of HRP on the surface post functionalization and validated successful immobilization of HRP-conjugated antibodies to the spinbar surface.

In addition to validating the attachment of antibodies to the surface of the spinbars, the ability of antibodies to remain functional post-conjugation was also verified via confocal microscopy (S1 Fig). Images showed GFP, which had been modified to include a His-tag, was retained on spinbars conjugated with anti-His antibodies but was not retained in the absence of the antibodies. This data demonstrated that functional antibodies had been conjugated to the surface of the spinbars using the stated methods.

To demonstrate the utility of Pyrex Spinbars for IMS, bioconjugation of spinbars was performed using antibodies to either 8X His or *Escherichia coli*. The antibody-conjugated spinbars were subsequently tested for their ability to capture his-tagged proteins or whole *E*.*coli* cells respectively from sample volumes equal to 0.5 L. Enhanced green fluorescent protein (EGFP) tagged with 6X His was isolated from crude cell lysates, whereas *E*. *coli* cells were isolated from a complex matrix consisting of a stomached ground beef slurry inoculated with *E*. *coli* O157:H7. SPPs with a surface area equivalent to the spinbars were also tested in these matrices for comparison purposes. Experimental controls consisted of solutions without EGFP or *E*. *coli* to define the background and spinbars/ SPPs that were devoid of antibodies to identify any non-specific binding that was occurring.

For EGFP capture, spinbars/ SPPs were mixed with lysates for 10 min and subsequently washed, with magnets being employed for particle movement during the recovery process. It’s worth noting that because of the time required for magnetic recovery of SPPs, both spinbars and SPPs remained in contact with the solution for an additional 30 min without mixing for experimental consistency. However, this additional residence time was not required for spinbars since their macroscopic size allows for rapid recovered from solutions; thus, shortening protocol duration. Post washing, EGFP was cleaved from the magnetic particles using an embedded protease site and the amount of captured EGFP was measured via fluorescence (Fig 2). This data demonstrated that the level of EGFP captured by the spinbar was essentially equivalent to that captured by the SPPs. It also demonstrated that use of either SPPs or spinbars would allow concentration of EGFP since the protein concentrations were statistically higher after IMS (Fig 2 versus Fig 2 inset).

The ability to capture larger targets (whole cells) by both the spinbars and SPPs was also tested using anti-*E*. *coli* conjugated spinbars/SPPs. In this assay, an additional level of matrix complexity was also added by using stomached ground beef inoculated with *E*. *coli* O157:H7 (10^5^ or 10^6^ cells/mL) as the matrix compared to cells lysed in buffer as was used prior for the capture of EGFP. To assess cell capture, quantitative real-time PCR (qPCR) was employed (Fig 3). These data demonstrated an increase in captured cells via spinbars as indicated by a decrease in cycle threshold values (Ct) compared to SPPs in this complex matrix and challenge the current dogma of using many monodispersed particles for IMS.

**Fig 4:**
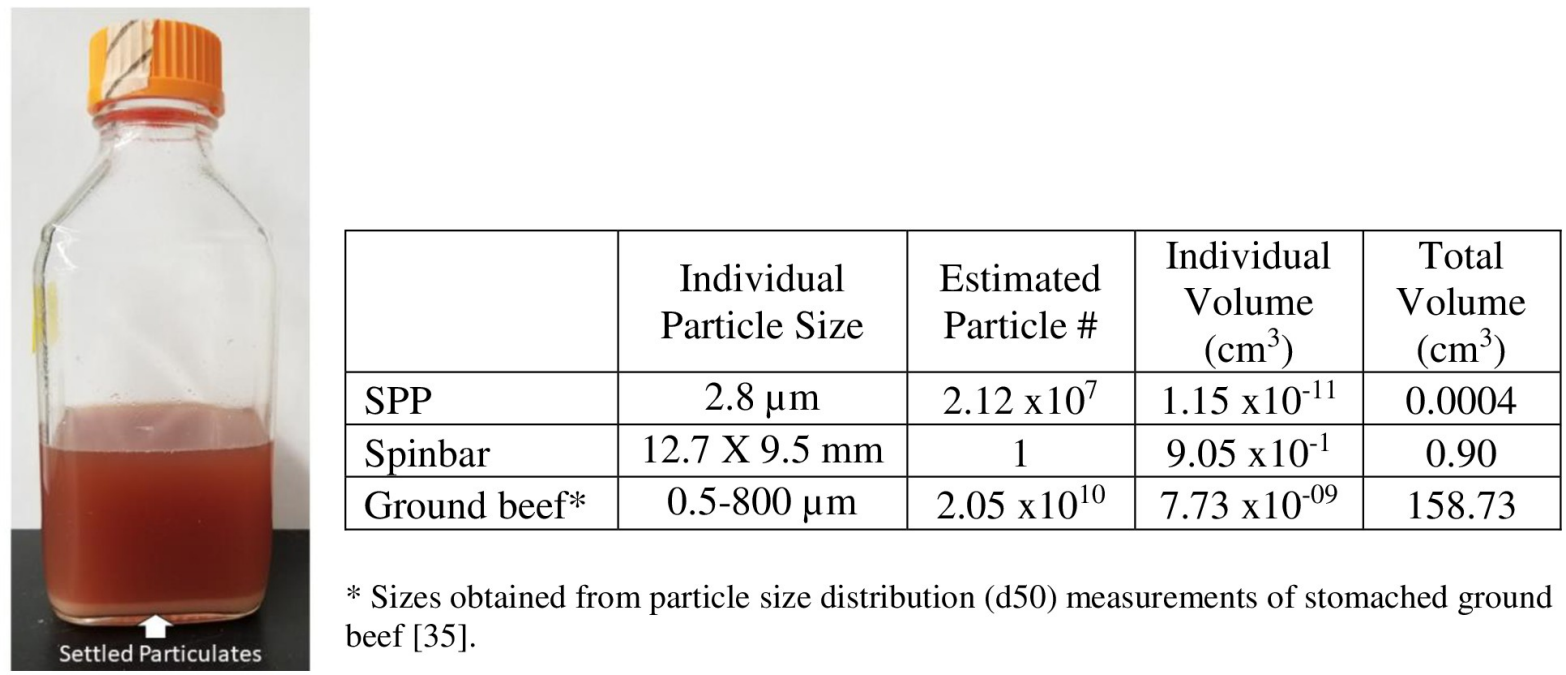
Particle recovery. Factors affecting particle recovery from stomached ground beef can include particulate settlement (left) as well as physical properties of both the magnetic particles and the matrix (right).

The lower level of captured cells via SPPs could be the result of either decreased capture by SPPs or a loss of SPPs during particle recovery since the magnetic recovery performance of SPPs are likely more sensitive to sample constituents than the spinbars. With spinbars, 100% recovery of the magnetic particle was achieved almost instantaneously because of their large size and net magnetic force. However, complete recovery of SPPs may be impeded by the ground beef matrix for several reasons (Fig 4). For instance, reduced magnetic recovery of SPPs is related to the volume of the beads relative to the volume of ground beef in the suspension as the ground beef presents a physical impediment for SPP movement. Entrapment of SPPs within the large particulate matter that can be found in ground beef [35] also increases the probability of accidental removal of those SPPs during the washing process. Targets such as pathogenic *E*. *coli* can further complicate the situation because they produce factors specifically designed to augment their ability to remain attached to host tissues [36], making separation from the matrix even more difficult. Furthermore, because the magnetic field strength decreases with increased distance between the SPP and the permanent magnet, recovery will be inversely proportional to volume of the sample [37].

Amplification of non-specific cell capture was also significantly reduced with spinbars as can be seen when comparing the capture of non-antibody coated spinbars against non-antibody coated SPPs (p = 0.0002), indicating cleaner sample preparations can be obtained with the spinbars. For example, when spinbars were employed, the capture rate of *E*. *coli* by the non-antibody coated spinbar controls could not be differentiated from that of the background controls devoid of *E*. *coli* O157:H7. This is contrary to the results obtained with the SPPs, where the Ct value for the non-antibody coated SPPs are lower than the Ct value for the SPPs devoid of *E*. *coli* O157:H7, indicating cells were captured regardless of the fact that no antibodies were present on the SPPs. This also implies that there may be less carryover of other matrix components as well when using spinbars compared to SPPs since spinbars lack the interstitial spaces that are present even with cubic/hexagonal close packing of hard spheres.

## Discussion

Methods involving the separation or isolation of a specified target from a heterogenous mixture are often utilized in research, diagnostic, and industrial applications. The effectiveness of a separation method typically depends not only upon the target but also the mixture that the target is present within. Techniques used for separation vary widely and take advantage of differences including but not limited to size, density, dielectrophoretic activity, and adherence. For example, filtration techniques separate target from non-target base upon size while centrifugation utilizes differences in density. Additionally, dielectrophoretic separation techniques are based upon the polarizability of cells, whereas adherence techniques rely upon the binding of the target to a solid support. Adherence techniques can take advantage of not only intrinsic binding abilities, such as the adherence of stromal cells from dental pulp to tissue culture plates [38], but also exploit the binding of antibodies to selected targets, such as the case with immunomagnetic separation [6].

Here, antibody-coated spinbars were shown to capture protein as well as cells from complex samples, thereby offering an additional means for the effective isolation of a target. As with other methods, the decision to use spinbars may be largely dependent upon the sample, type of target to be isolated, and its abundance in the sample. For example, if complete capture of a target that is in high abundance in a relatively refined sample is desired, and assay time is not a factor, the increased surface area that can be obtained using SPPs may prove beneficial for this application. Although use of a larger spinbar could also increase the area of available capture surface, the increase is not as remarkable. Also, spinbars should not be employed unless there is adequate sample volume to coat the entire surface of the spinbar. Hence, if the sample volume is small SPPs should be used. Contrary to that, when processing large-volume samples or heterogenous mixtures, such as stomached ground beef, spinbars may be more favorable. One reason for this is because mixing can be simplified with spinbars since many different types of samples can be effectively mixed with ease using a common laboratory magnetic stir plate. Generally, two main types of fluidic-flow patterns exist (laminar versus turbulent flow). Mixing via laminar flow is less effective because the liquid layers simply flow over one another whereas in turbulent flow, the layers distort and mixing occurs in both vertical and lateral dimensions [39]. Stir plates can produce both types of flow patterns depending upon the speeds employed [39], making manipulation of this factor straightforward with spinbars. In contrast, common agitation methods with SPPs involve inversion of the entire sample, which becomes cumbersome or even impractical with larger volumes.

The mixing that occurs when using a stir plate also differs from that produced by inversion. Stir plates mix by rotating the spinbar within the sample, which creates a downward sweep flow pattern [40]. This downward flow pattern has the advantage of pulling the target down onto the capture surface of the spinbar. This is in contrast to methods used with SPPs, which rely upon widespread distribution of the SPPs throughout the sample to facilitate SPP-target interactions. Despite the ability of the spinbar to simplify some aspects of the mixing process for IMS, many factors remain that govern one’s ability to effectively mix a sample. Such factors and their interactions would need to be accounted for during method optimization for a specified sample type or during process scaling and include but are not limited to vessel size, vessel shape, sample volume, spinbar size, spinbar shape, etc.

Target separation also plays a role in the effectiveness of any magnetic capture device. Target separation, in the context of IMS, is the product of the capture element employed and one’s ability to partition the magnetic particle(s) away from the matrix. Changes to the capture element, such as the use of antibodies with increased target affinity, may improve target capture and the overall capacity to recover the target. The influence of the capture element on target separation is equivalent for the spinbars and SPPs if the same capture element is used. However, magnetic separation of the particle(s) can differ drastically since it is dependent upon a multitude of factors including magnetic forces, hydrodynamic drag, and gravity [41]. For effective separation to occur with any sample, the magnetic forces must overcome the opposing forces such as hydrodynamic drag and gravity. For finer particles, the separation efficiency is more likely to collapse as the liquid drag force becomes greater than the magnetic force exerted between the magnetic separator and the magnetic particles [42]. Pyrex spinbars typically contain an Alnico magnet encapsulated in borosilicate glass. In contrast to the negligible remanence of SPPs, the remanence of Alnico is high. The larger size of the spinbar also allows more magnetic material to be incorporated compared to SPPs. Together, these design factors increase the magnetic forces and thereby enhance the recovery rate while simultaneously decreasing the recovery time for the spinbars. This ultimately results in shortened protocol times when spinbars are employed.

The macroscopic size of the spinbar offers additional advantages as well as disadvantages compared to microscopic-sized particles. First, its size allows visual confirmation that it was recovered from the solution. Its large size would also prevent the spinbar from being internalized by target cells and therefore, eliminate any toxic effects associated with that internalization as has been previously reported with cell capture [43]. However, the large size of the spinbar can be disadvantageous as well. One disadvantage of the spinbar’s size is that captured cells must be removed from the spinbar prior to plating onto solid media. Unlike SPPS, whose minute size allows any attached cells to be in contact the surface of an agar plate, only a fraction of the spinbar can come into contact with the planar surface of the agar plate at a given time. This prevents the simultaneous growth of all of the cells captured unless placed into a liquid medium. To overcome this disadvantage, there are commercial kits available aimed at detaching cells from beads that would likely be applicable to the spinbar as well [7]. The spinbar is also not applicable to small sample volumes because it requires a greater volume of liquid compared to SPPs to cover its surface and ensure exposure of the capture elements to targets within the sample.

Overall, purification of protein or cells from large-volume, complex matrices has far-reaching implications in fields such as biopharmaceuticals, environmental monitoring, and diagnostic microbiology [6, 7]. Benefits of using spinbars for IMS include an improved particle retrieval process yielding an almost instantaneous 100% particle recovery, an easy yet effective mixing strategy, reduced transfer of sample matrix, and the capacity to handle large volumes of complex samples. Spinbars also remain adaptable to automation based on their magnetic properties, are capable of being manufactured in a range of sizes for scale-up purposes, and may be coated with inexpensive chemical solutions or vapor deposition. Considering the presented method would be amendable to capture alternative proteins and target cells and is likely effective for capturing DNA targets through the incorporation of oligonucleotide-coatings; spinbars not only provide a viable alternative to SPPs but can also expand the utility of IMS.

## Supporting information

S1 Fig Verification of antibody immobilization on spinbars. Spinbars conjugated with anti-His antibodies were used to capture purified green fluorescent protein (GFP). Images showing capture of the GFP by conjugated spinbars were taken under 40X magnification using a confocal microscope (left). Spinbars not exposed to GFP were used as controls (right) with images captured under identical conditions. Scalebars are shown in the lower left corner.
(TIF)
