# Supplementary figures and images for: Magnetic capture device for large volume sample analysis
Source: PLoS One. 2024 Feb 9;19(2):e0297806. doi: 10.1371/journal.pone.0297806 (PMC10857679; doi:10.1371/journal.pone.0297806)

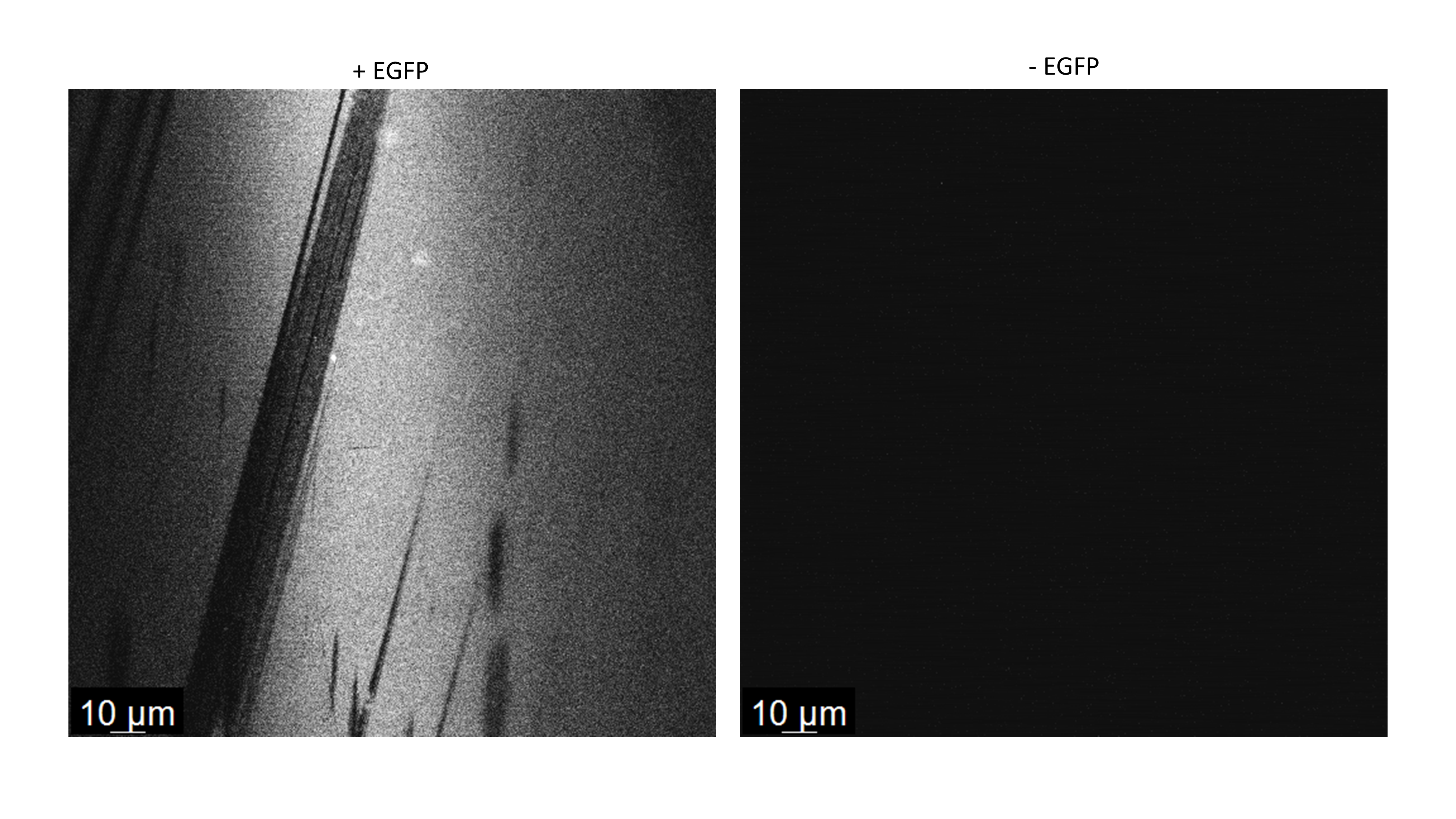

Supplement: S1 Fig — Spinbars conjugated with anti-His antibodies were used to capture purified green fluorescent protein (GFP). Images showing capture of the GFP by conjugated spinbars were taken under 40X magnification using a confocal microscope (left). Spinbars not exposed to GFP were used as controls (right) with images captured under identical conditions. Scalebars are shown in the lower left corner. (TIF) [file pone.0297806.s001.tif]
